# Correction: Feng et al. Electrolyte Analysis in Blood Serum by Laser-Induced Breakdown Spectroscopy Using a Portable Laser. *Molecules* 2022, *27*, 6438

**DOI:** 10.3390/molecules29163771

**Published:** 2024-08-09

**Authors:** Zhongqi Feng, Shuaishuai Li, Tianyu Gu, Xiaofei Zhou, Zixu Zhang, Zhifu Yang, Jiajia Hou, Jiangfeng Zhu, Dacheng Zhang

**Affiliations:** 1 School of Optoelectronic Engineering, Xidian University, Xi’an 710071, China; zhongqifeng@stu.xidian.edu.cn (Z.F.); 20051212253@stu.xidian.edu.cn (S.L.); tygu@stu.xidian.edu.cn (T.G.); zhangzixv@foxmail.com (Z.Z.); houjj@xidian.edu.cn (J.H.); jfzhu@xidian.edu.cn (J.Z.); 2 Clinical Laboratory, The Hospital of Xidian University, Xi’an 710071, China; zhouxfzyh712@163.com; 3 Department of Pharmacy, Xijing Hospital, Xi’an 710032, China

## Error in Figure

In the original publication [1], there was a mistake in Figure 6. The quantitative analysis of elements in blood serum on filter paper. (a) K; (b) Na; (c) Ca. as published. The Figure 6b,c have been duplicated in error. The corrected Figure 6. The quantitative analysis of elements in blood serum on filter paper. (a) K; (b) Na; (c) Ca. appears below. The authors state that the scientific conclusions are unaffected. This correction was approved by the Academic Editor. The original publication has also been updated.

## Figures and Tables

**Figure 6:**
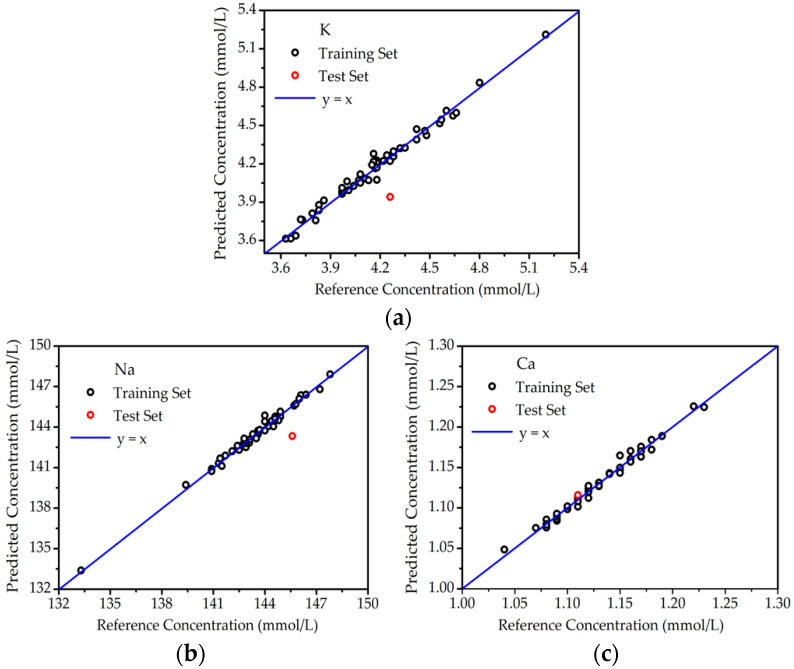
The quantitative analysis of elements in blood serum on filter paper. (**a**) K; (**b**) Na; (**c**) Ca.
